# Supplementary material for: Post-translational regulation of lipogenesis via AMPK-dependent phosphorylation of insulin-induced gene
Source: Nat Commun. 2019 Feb 7;10:623. doi: 10.1038/s41467-019-08585-4 (PMC6367348; doi:10.1038/s41467-019-08585-4)
Supplement: Supplementary file 1 — Supplementary Information [file 41467_2019_8585_MOESM1_ESM.pdf]

Supplementary Information

Post-translational regulation of lipogenesis via AMPK phosphorylation of insulin-induced gene

Han et al.

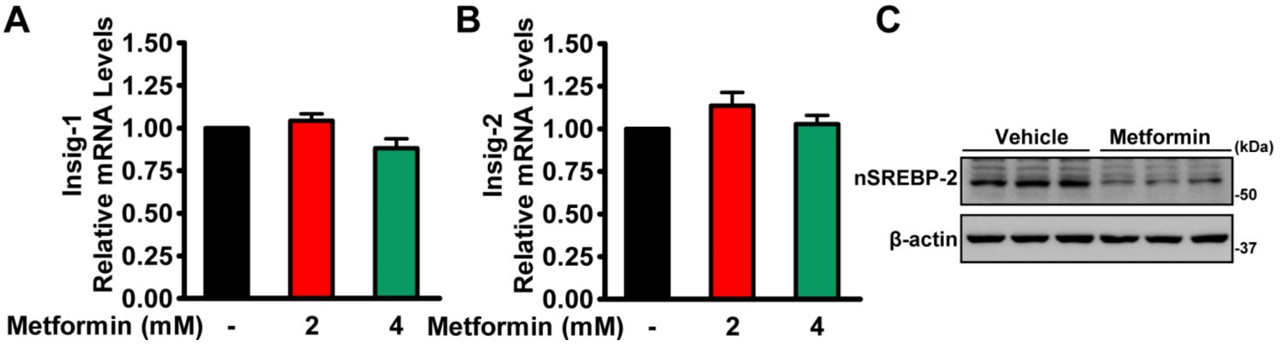

Supplementary Figure 1. Effects of metformin on the mRNA levels of Insig-1 and Insig-2. (A-B) HEK293 cells were treated with various doses of metformin for 24h. Real-time PCR analysis was performed to measure mRNA levels of Insig-1 (A) and Insig-2 (B). The data are represented as the mean±SEM, n=3. (C) AMPK activation by metformin decreases the cleavage processing of SREBP-2 in the liver of mice fed with high-fat, high-sucrose (HFHS) diet.

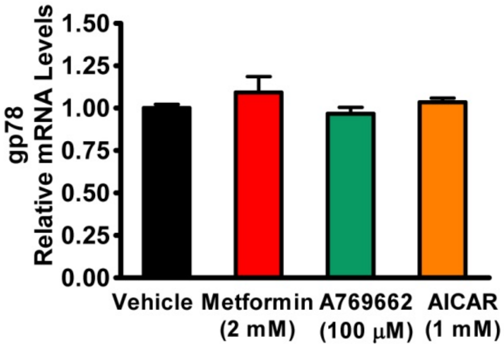

Supplementary Figure 2. Effects of AMPK agonists on the expression of gp78. HEK293 cells were treated with 2 mM metformin, 100 μM A769662, or 1 mM AICAR. mRNA levels of gp78 were measured by real-time PCR. The data are represented as the mean±SEM，n=3.

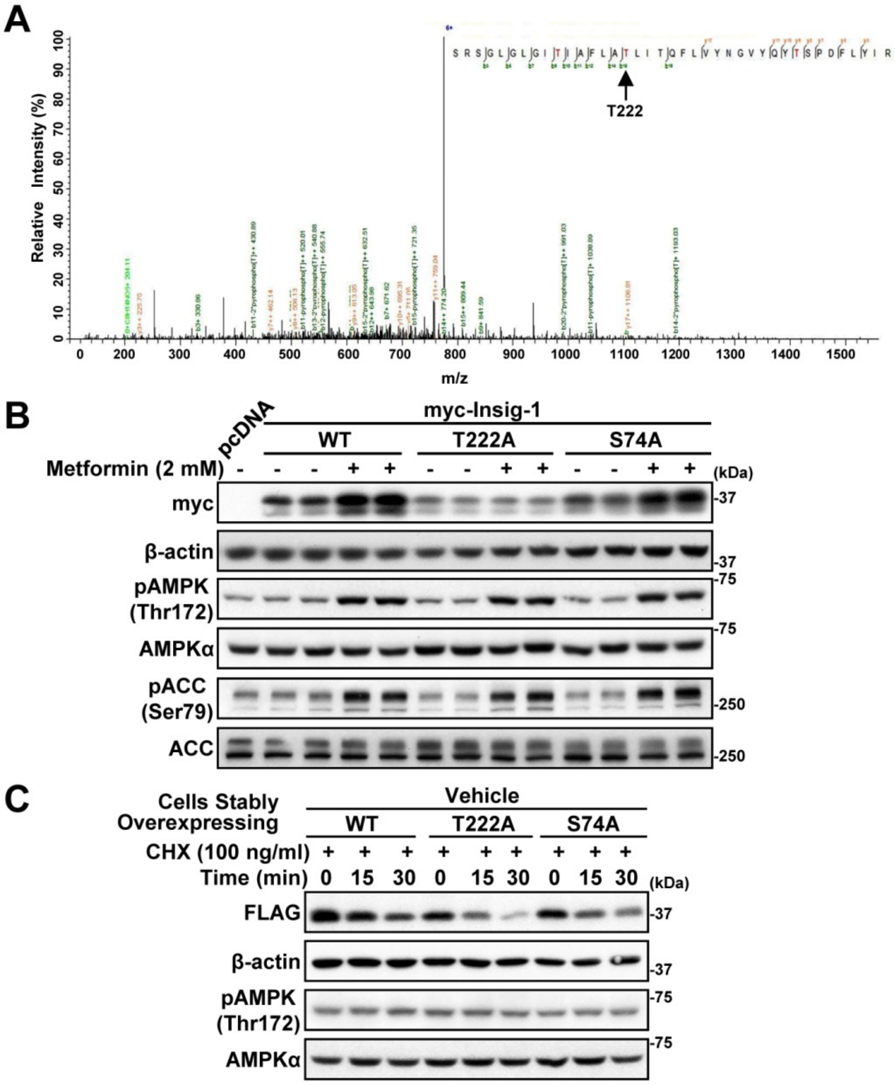

Supplementary Figure 3. AMPK phosphorylates Insig-1 at Thr222 residues. (A) Phosphorylation of T222 was demonstrated by liquid chromatography-tandem mass spectrometry (LC-MS/MS) analysis showing phosphorylated Thr222 residue of human Insig-1 protein. HEK293 cells were transfected with myc-tagged Insig-1, followed by treatment with A769662 for 8 h. The cell lysates were used for proteomic analysis. (B) Thr222 site is required for metformin-induced Insig-1 in HEK293 cells. Cells were transfected with pcDNA or myc-tagged Insig-1 WT and nonphosphorylatable S74A or T222A mutant plasmids for 24 h, followed by treatment with 2 mM metformin for 24 h. Immunoblots were performed. (C) The effects of cycloheximide-induced degradation of Insig-1 and its nonphosphorylatable mutants. HEK293 cells stabling expressing FLAG-tagged Insig-1 WT, T222A or S74A were treated with Vehicle (PBS), followed by incubation in the medium containing 100 ng cycloheximide for the indicated time.

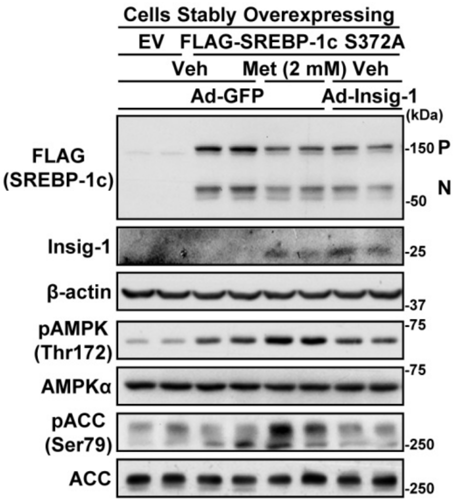

Supplementary Figure 4. Metformin-increased cleavage processing of SREBP-1c is likely via stabilization of Insig-1. HepG2 cells stably expressing SREBP-1c S372 mutant was infected with low-dose of adenoviruses encoding Insig-1 (Ad-Insig-1) or GFP for 48 h, followed by treatment with 2 mM metformin for 24 h.

Supplementary Figure 5. Uncropped western blot images of the indicated Figures.

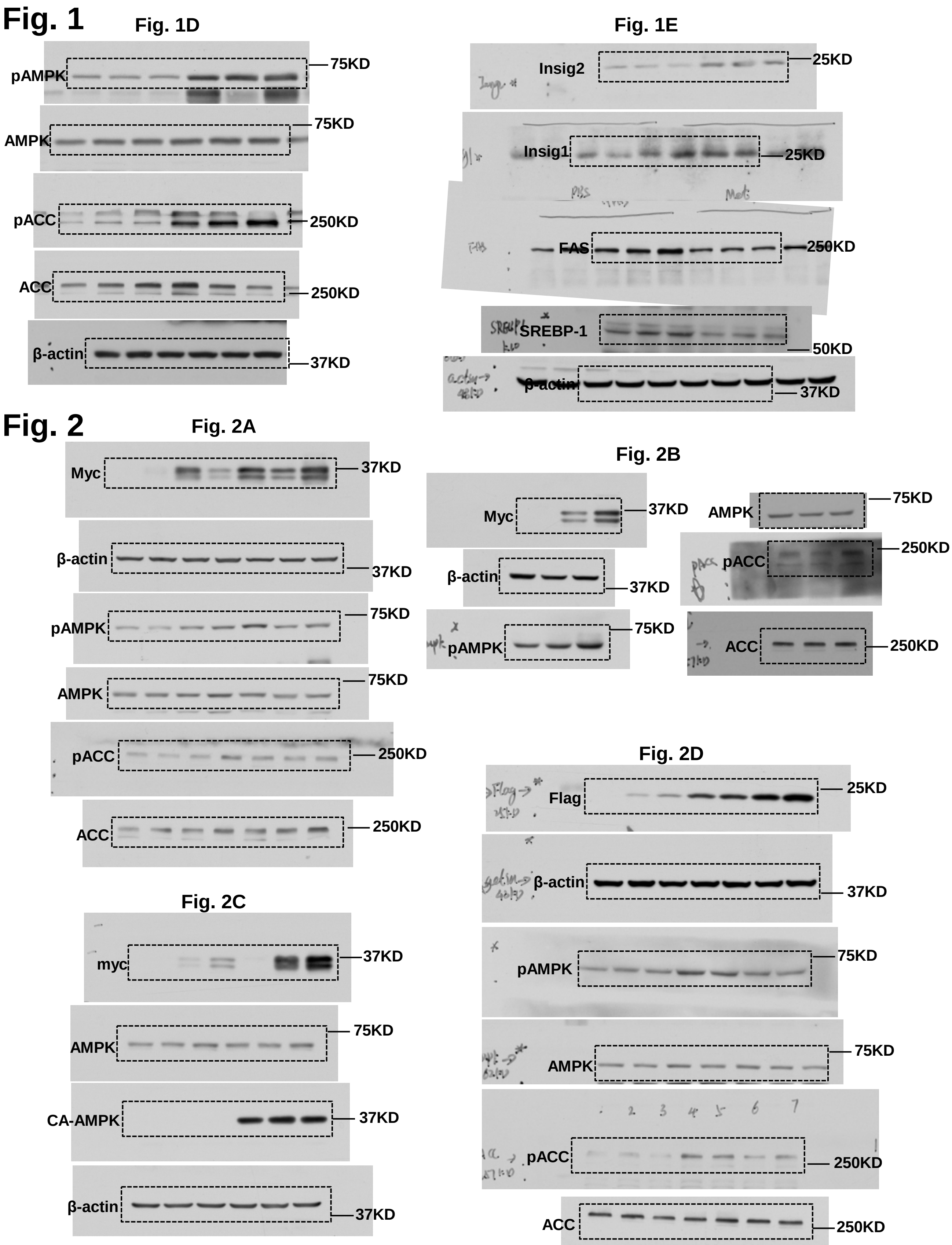

Fig. 2

Fig. 2E

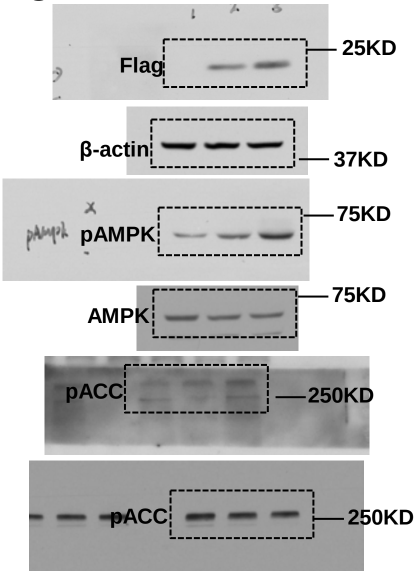

Fig. 2F

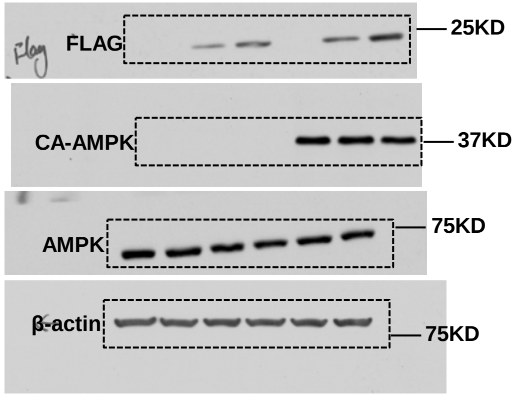

Fig. 3

Fig. 3A

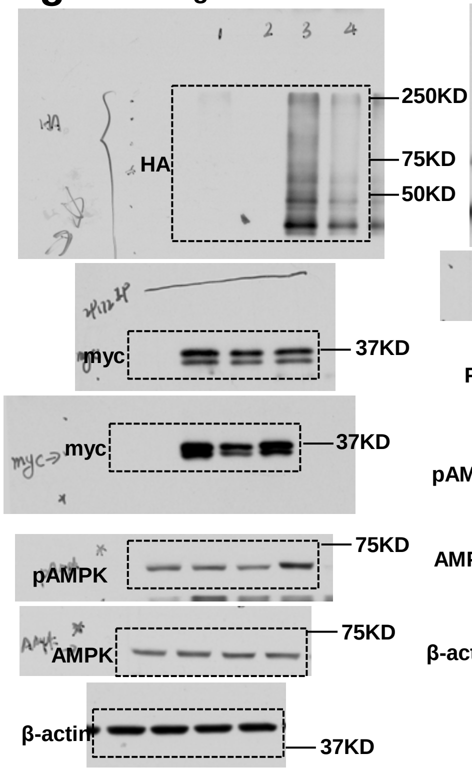

Fig. 3B

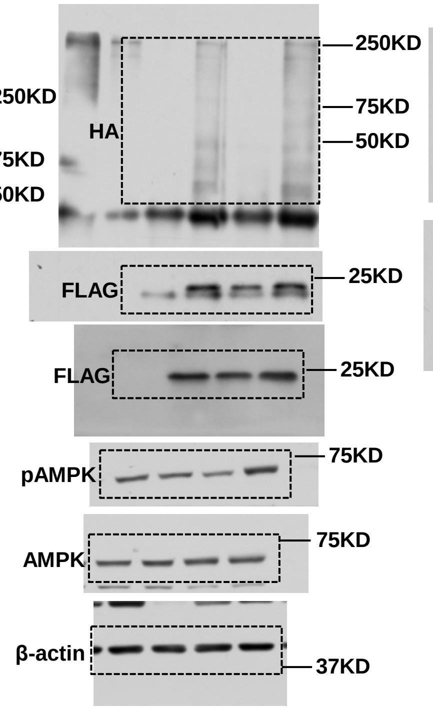

Fig. 3C

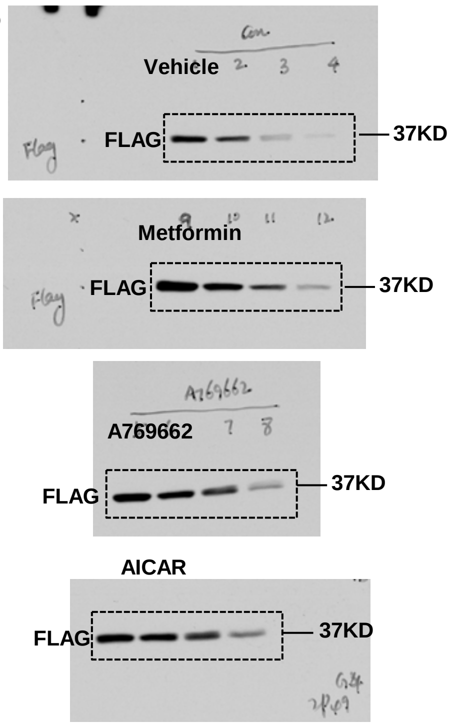

Fig. 4

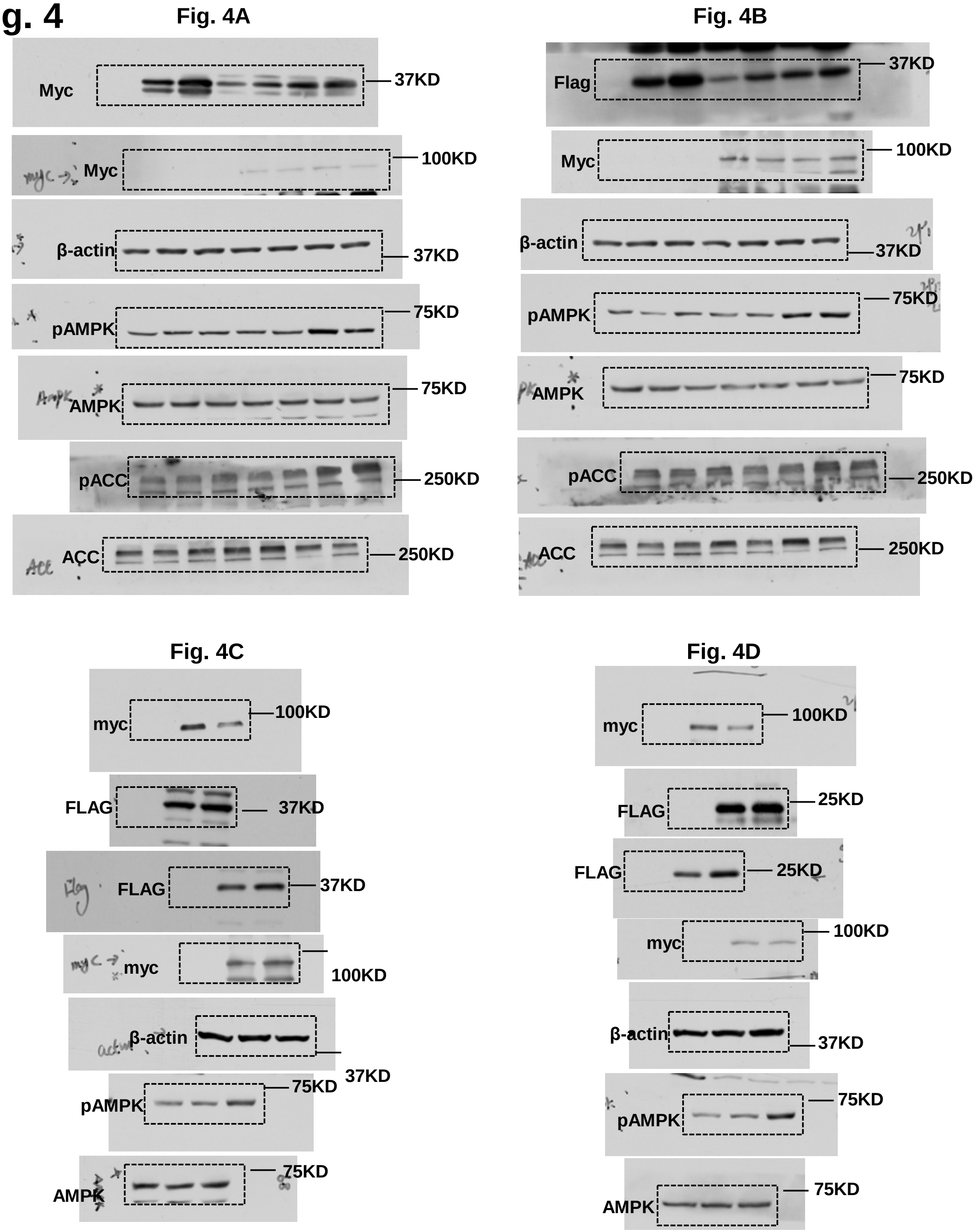

Fig. 4A
Myc
37KD
Myc
100KD
β-actin
37KD
pAMPK
75KD
AMPK
75KD
pACC
250KD
ACC
250KD
Fig. 4B
Flag
37KD
Myc
100KD
β-actin
37KD
pAMPK
75KD
AMPK
75KD
pACC
250KD
ACC
250KD
Fig. 4C
myc
100KD
FLAG
37KD
FLAG
37KD
myc
100KD
β-actin
37KD
pAMPK
75KD
AMPK
75KD
Fig. 4D
myc
100KD
FLAG
25KD
FLAG
25KD
myc
100KD
β-actin
37KD
pAMPK
75KD
AMPK
75KD

Fig. 5

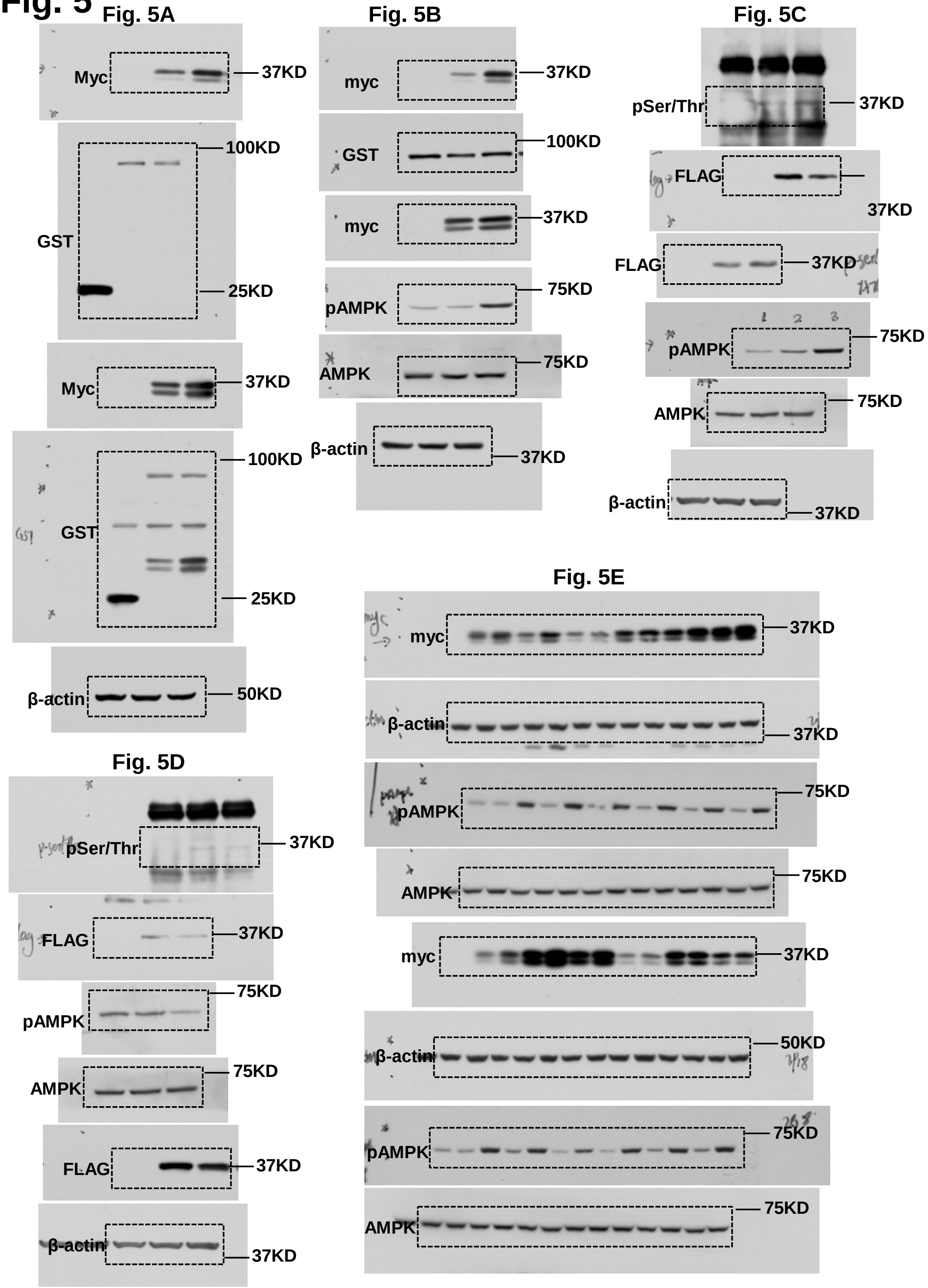

Fig. 5A
Myc
37KD
100KD
GST
25KD
Myc
37KD
100KD
GST
25KD
β-actin
50KD
Fig. 5B
myc
37KD
GST
100KD
myc
37KD
pAMPK
75KD
AMPK
75KD
β-actin
37KD
Fig. 5C
pSer/Thr
37KD
FLAG
37KD
FLAG
37KD
pAMPK
75KD
AMPK
75KD
β-actin
37KD
Fig. 5D
pSer/Thr
37KD
FLAG
37KD
pAMPK
75KD
AMPK
75KD
FLAG
37KD
β-actin
37KD
Fig. 5E
myc
37KD
β-actin
37KD
pAMPK
75KD
AMPK
75KD
myc
37KD
β-actin
50KD
pAMPK
75KD
AMPK
75KD

Fig. 5

Fig. 5F

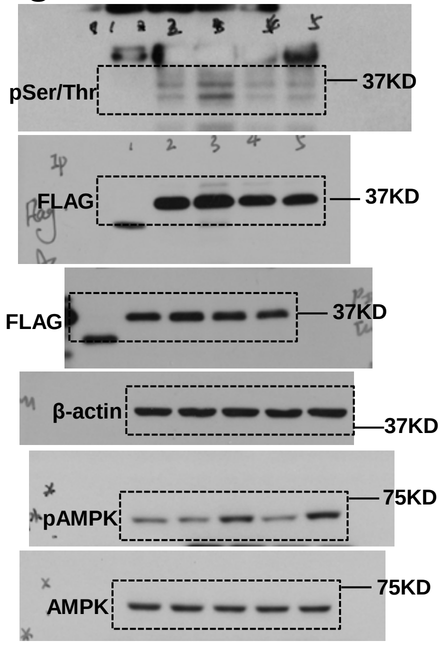

Fig. 5G

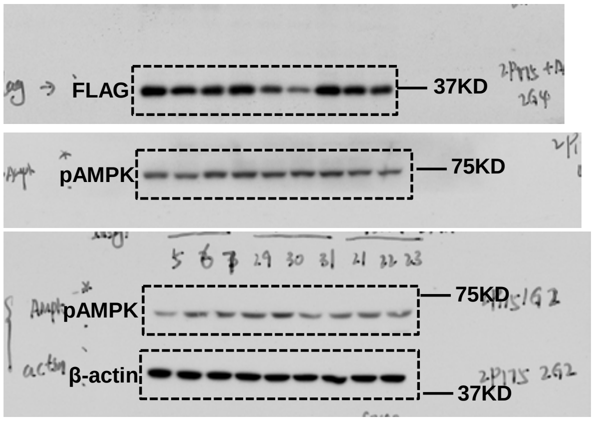

Fig. 6

Fig. 6A

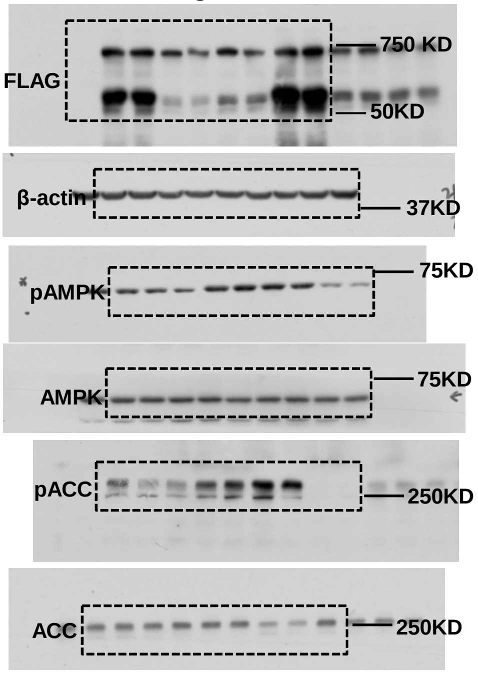

Fig. 6C

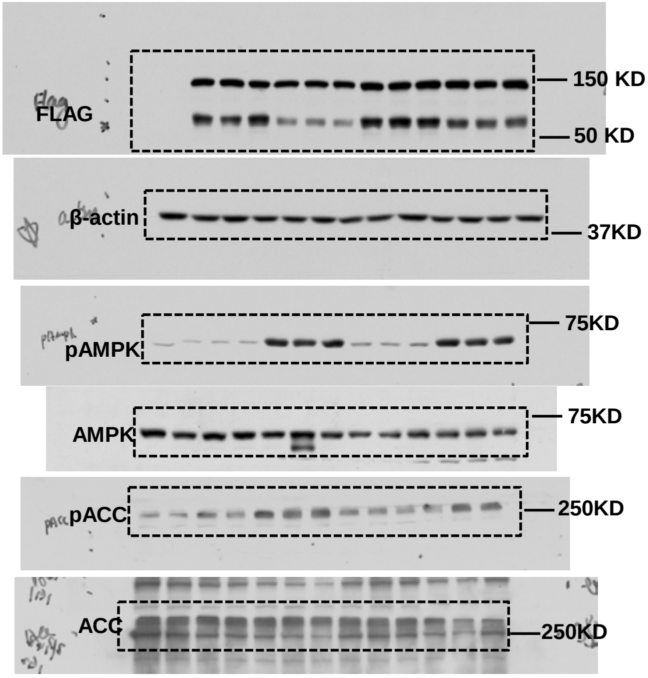

Fig. 6B

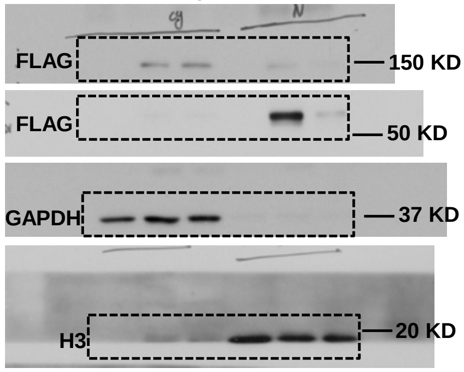

Fig. 6E

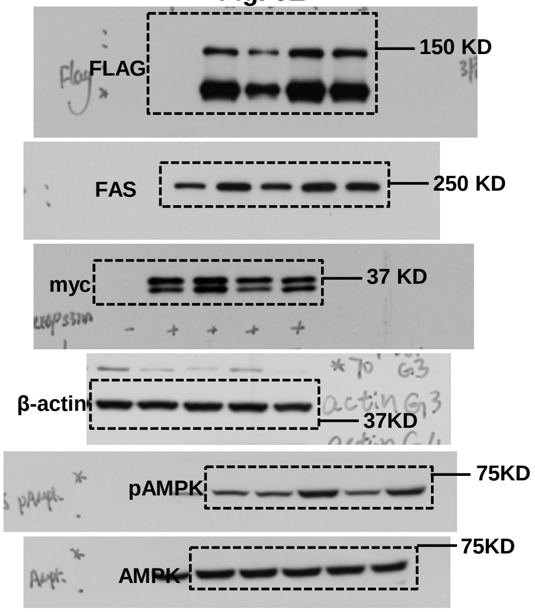

Fig. 7

Fig. 7A

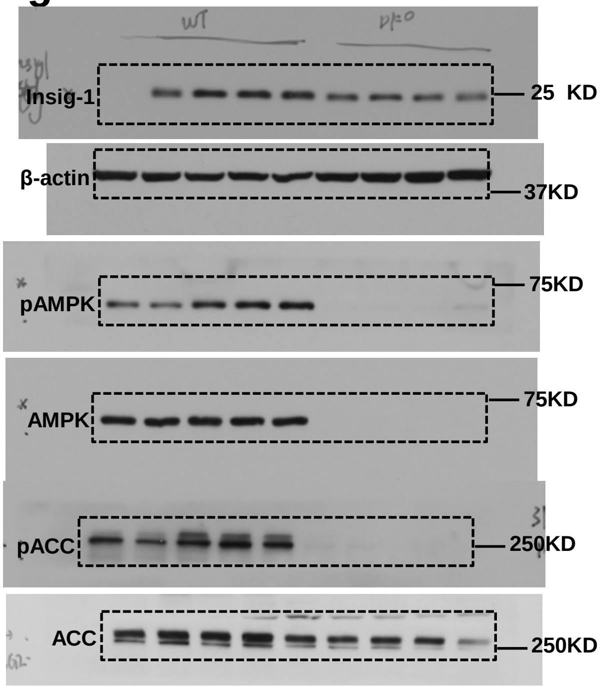

Fig. 7B

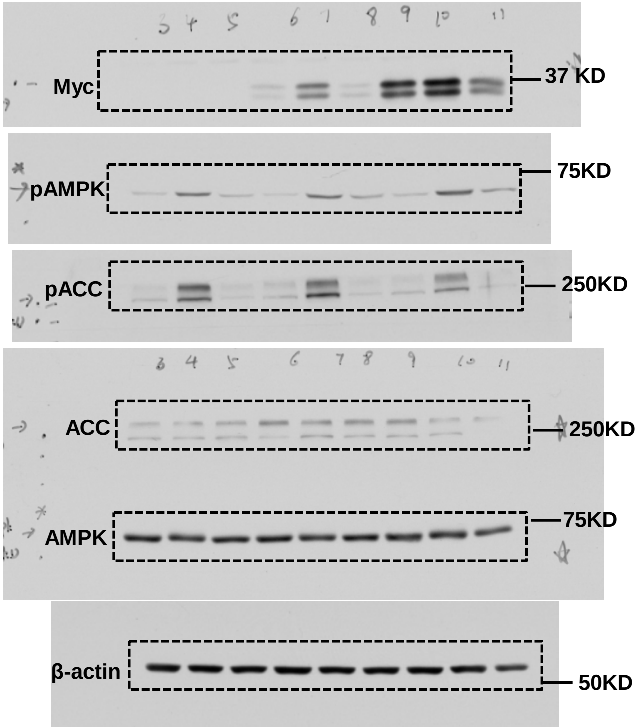

Fig. 7C

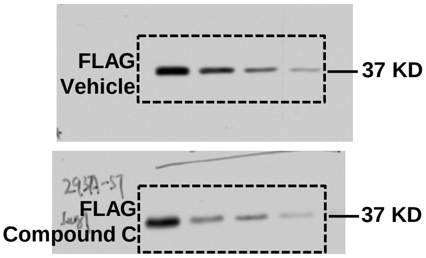

Fig. 7D

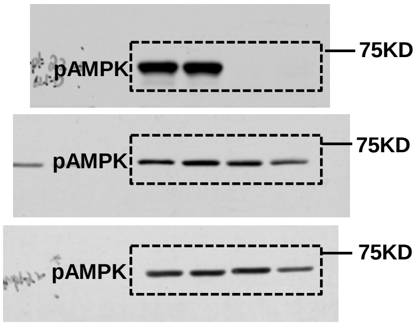

Fig. S1

Fig. S1C

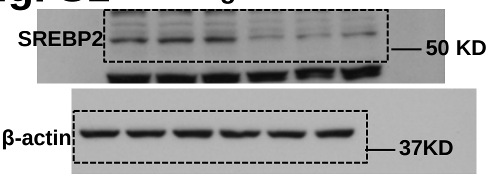

Fig. S3

Fig. S3B

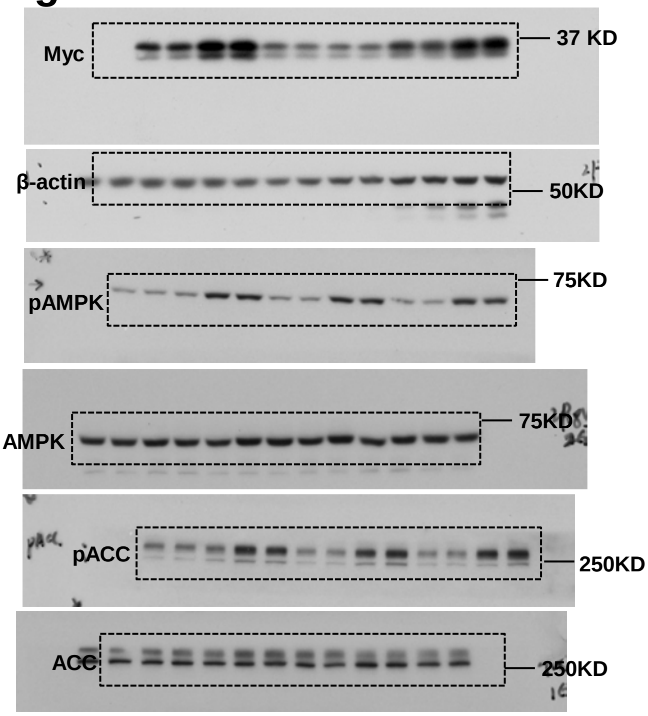

Fig. S3C

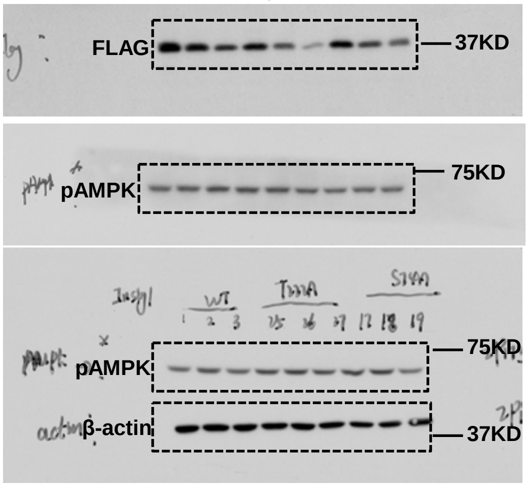

Fig. S4

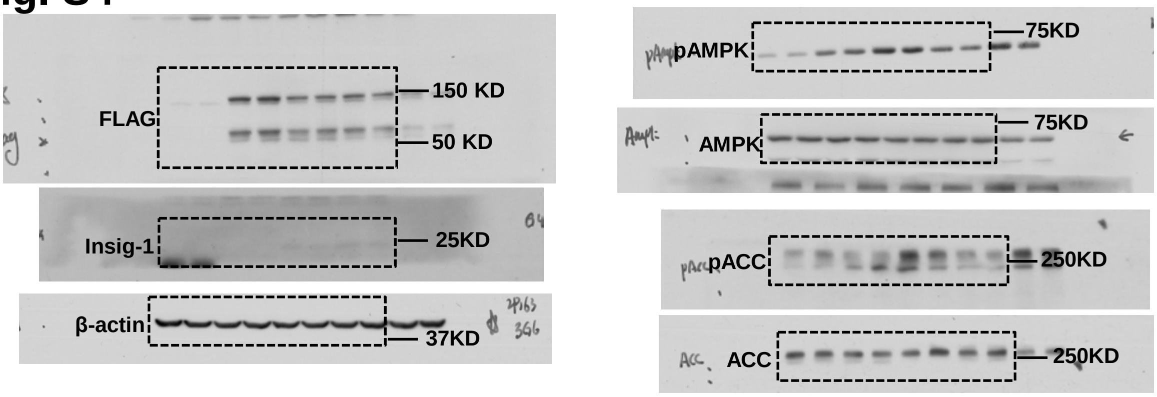

Supplementary Table 1. Quantitative RT-PCR primers

Gene	Species	Forward primer	Reverse primer
Insig-1	human	TTACACCCTCCACTGAACCC	CATTCTGCTGACTGGTGGC
Insig-1	human	CTGCTGTTGTTGGCCTACTG	TGACACTGGCCCATTCTCTC
Insig-2	human	CCACAAGGGCAAGATCTTCTG	CACTCACTCCAACACACACG
β-actin	human	GATGAGATTGGCATGGCTTT	GTCACCTTCACCGTTCCAGT
FAS	mouse	GCTGCGGAAACTTCAGGAAAT	AGAGACGTGTCACTCCTGGACTT
SREBP-1c	mouse	GGAGCCATGGATTGCACATT	GGCCCGGGAAGTCACTGT
ACC1	mouse	TGACAGACTGATCGCAGAGAAAG	TGGAGAGCCCCACACACA
SCD1	mouse	TTCTTCTCTCACGTGGGTTG	CGGGCTTGTAGTACCTCCTC
ACLY	mouse	GCCAGCGGGAGCACATC	CTTTGCAGGTGCCACTTCATC
GPAT	mouse	CAACACCATCCCCGACATC	GTGACCTTCGATTATGCGATCA
DGAT-1	mouse	GATTGTGGGCCGATTCTTCC	CATACATGAGCACAGCCACC
DGAT-2	mouse	GGTTATCTCGCTGCTGTTGG	TGTGGCTCAGGAGGATATGC
β-actin	mouse	CCACAGCTGAGAGGGAAATC	AAGGAAGGCTGGAAAAGAGC
